# Analysis of biomechanical behavior of 3D printed mandibular graft with porous scaffold structure designed by topological optimization

**DOI:** 10.1186/s41205-019-0042-2

**Published:** 2019-03-14

**Authors:** Jiajie Hu, Joanne H. Wang, Russel Wang, Xiong Bill Yu, Yunfeng Liu, Dale A. Baur

**Affiliations:** 1 Department of Electrical Engineering and Computer Science, Case Western Reserve University School of Engineering, Cleveland, OH 44106-7201 USA; 2 Department of Orthopedic Surgery, Case Medical Center, Cleveland, OH 44106 USA; 3 Department of Comprehensive Care, Case Western Reserve University School of Dental Medicine, Cleveland, OH USA; 4 Department of Civil Engineering, Case Western Reserve University School of Engineering, Cleveland, OH 44106-7201 USA; 5 Key Laboratory of E&M (Zhejiang University of Technology), Ministry of Education & Zhejiang Province, Hangzhou, 310014 Zhejiang Province China; 6 Department of Maxillofacial Surgery, Case Western Reserve University School of Dental Medicine, Cleveland, OH 44106 USA

**Keywords:** 3D printing, Anisotropic behavior, Finite element analysis, Fused deposition modeling, Mandibular graft, Poly-lactic acid, Topological optimization

## Abstract

**Background:**

Our long-term goal is to design and manufacture a customized graft with porous scaffold structure for repairing large mandibular defects using topological optimization and 3D printing technology. The purpose of this study is to characterize the mechanical behavior of 3D printed anisotropic scaffolds as bone analogs by fused deposition modeling (FDM).

**Methods:**

Cone beam computed tomography (CBCT) images were used to reconstruct a 3D mandible and finite element models. A virtual sectioned-block of the mandible was used as the control group and the trabecular portion of the block was modified by topological optimization methods as experimental groups. FDM (FDM) printed samples at 0, 45 and 90 degrees with Poly-lactic acid (PLA) material under a three-point bending test. Finite element analysis was also used to validate the data obtained from the physical model tests.

**Results:**

The ultimate load, yield load, failure deflection, yield deflection, stress, strain distribution, and porosity of scaffold structures were compared. The results show that the topological optimized graft had the best mechanical properties.

**Conclusions:**

The results from mechanical tests on physical models and numerical simulations from this study show a great potential for topological optimization and 3D printing technology to be served in design and rapidly manufacturing of artificial porous grafts.

## Introduction

Autograft and allograft are commonly used for bone grafting procedures to repair segmental bony defects, which usually result from trauma, infection and tumor resection of tumors [1–3]. The limitations of autografts are donor site morbidity [4], lack of bone volume for a large defect [5, 6], and possible nerve damage. While allografts are limited by anatomical variations, genetic differences, and possible disease transmission [7, 8]. It is important to restore a critical-sized mandibular defect to its original size and shape to achieve desirable facial esthetics and functional outcome for subsequent prosthetic reconstruction. Microvascular free fibular graft is a common method for mandibular reconstructions to repair a large segmental defect [9–12]. It is impossible of using a fibular bone to match the shape and size of the resected portion of a mandible. Therefore, there are many prosthetic complications for those patients who received surgical and prosthetic reconstruction of the mandible [13]. One promising approach for obviating the aforementioned complication is the implementation of 3D printing strategies for the manufacturing of customized artificial bio-graft [14, 15].

3D printing is a new digital modeling technology that emerged in the late 1980s. The core processing principle is that based on the principle of layer-by-layer overlay printing. The printing material could accurately be stacked to any shape of 3D complex objects in the control of computer programs [16, 17]. In recent years, more and more attention has been paid to research and development of 3D printing technology in medicine. 3D printing is used to create objects with complex shapes and form. 3D “bioprinting” [18, 19] has a great potential for the application of using biomaterials to print bony defects for mandibular reconstructions resulting from tumor resection and trauma. In some cases, researchers can use 3D printing technology to produce personalized biomaterials that meet clinic needs. Schieker et al. [20] used 3D printing to create models with intricate internal structures with great precision in the range of micrometer level. The aperture size was as low as 450 μm and the wall thickness was 330 μm. The mechanical strength of the structure was more than 22 MPa, which can meet the general requirements of an organ model. Yang et al. [21] used nano-hydroxyapatite and polyester microspheres to form polyester and nano-hydroxyapatite composites by 3D printing for medical applications. Habibovic et al. [22] demonstrated that complex shapes of implants could be 3D printed using calcium hydroxide phosphate and montmorillonite composites at body temperature with good mechanical properties for clinical use. The implanted fillers made from the two materials have varying degrees of osteoconduction and osteoinduction. However, studies found that there are significant changes of the mechanical properties resulting from anisotropy in 3D printing. Fused deposition modeling (FDM) has been widely used in all fields. Poly-Lactic Acid (PLA) is a commonly used material for 3D printing. [23] Because of the layer-by-layer manufacturing procedure, the model processed by FDM has a layered orthotropic microstructure, in which each layer consists of a contour and infill strips [24–26]. The infill structures may have different patterns depending on the printers.

Our long-term goal is to develop a new method to overcome the drawbacks of traditional bone grafting procedures for treating segmental bony defects by using 3D printing technology. 3D virtual mandibular grafts were designed based on cone beam computed tomography (CBCT) images and subsequently fabricated by 3D FDM printing for reconstruction of a patient-specific mandibular defect. PLA is a promising thermoplastic aliphatic polyester and has been extensive studies for biomedical applications. It has been proven to be safe in clinical use such as temporary and long-term implantable devices, tissue engineering, and drug delivery systems [27–33]. PLA is a biocompatible material and its biodegradation in vivo is through hydrolysis to convert to water and carbon dioxide. The biodegradation behavior is a critical characteristic of the materials and the most important reason for the high interest in its use in medical applications and industry. It begins to decompose into lactic acid (LA), carbon dioxide and water once in contact with biological media. These products are metabolized in cells or excreted in urine and respiration. The 3D printing of customized grafts consist of the original shape and form of the cortical layer of a segmented mandibular with internal porous scaffold. The internal scaffolds had different strut designs to give strength during masticatory function. Topological optimization process was performed to provide an efficient way for the reduction of structure volume and the improvement of strength and stability [30–33]. Biomechanical behaviors of three types of grafts were compared and analyzed. The maximum load, yield load, failure deflection and yield deflection were measured from a three-point bending test for each type of the printed grafts. Maximum von Mises stress, principal strain, and displacement were calculated from finite element analysis using numerical simulation models.

## Materials & methods

### CBCT image, 3D reconstruction and meshing

**Fig. 1:**
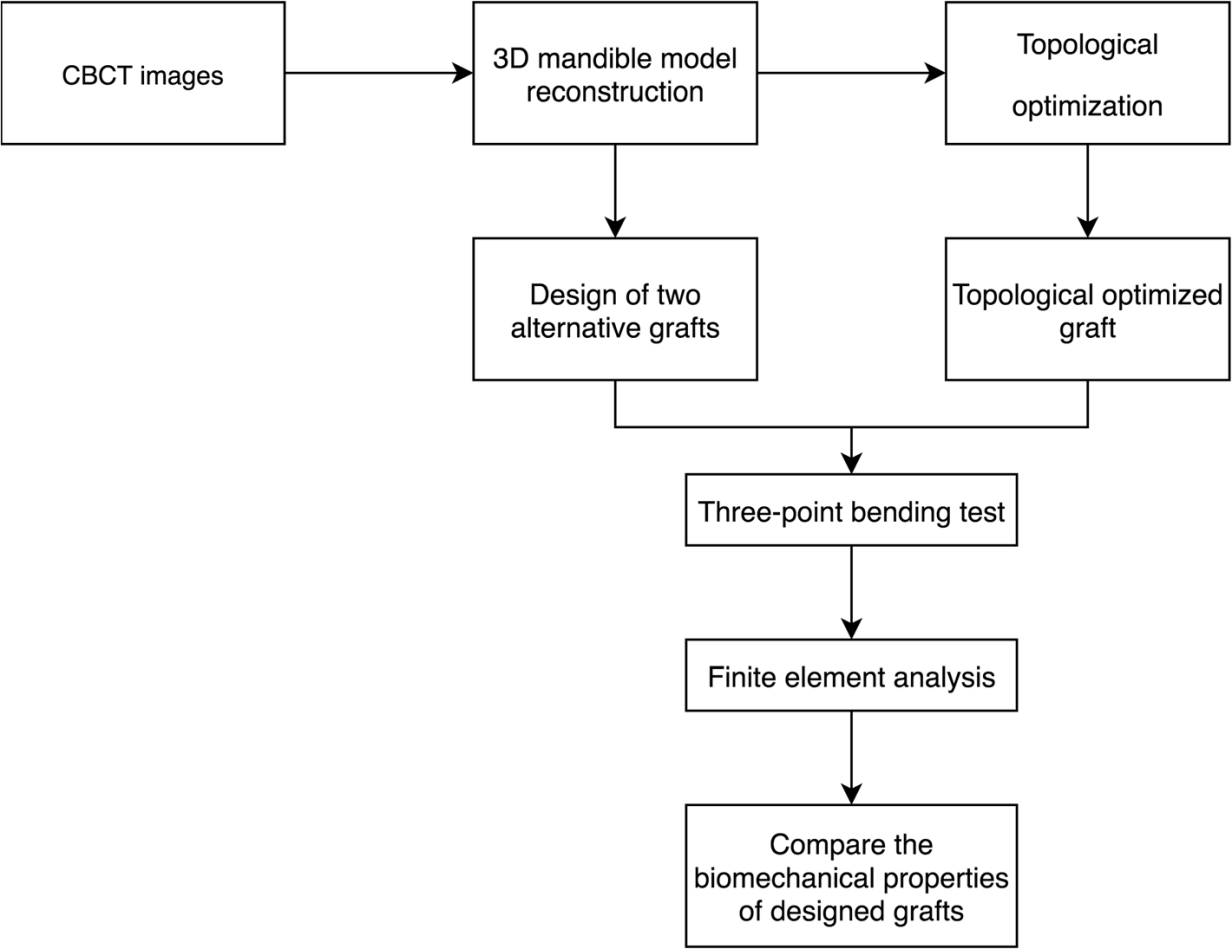
Flowchart of the study

CBCT has been widely used in treatment planning and diagnosis in implant dentistry [34–37]. Digital image data were obtained through a cone beam CT scanner (120 K 70 mA) with field of view of 23 × 16 cm and voxel size of 0.39 mm from a 50-year-old edentulous patient without any pathological condition in the mandible. A total of 512 images were saved as DICOM data files. Figure 1 shows the flowchart of this study.

**Fig. 2:**
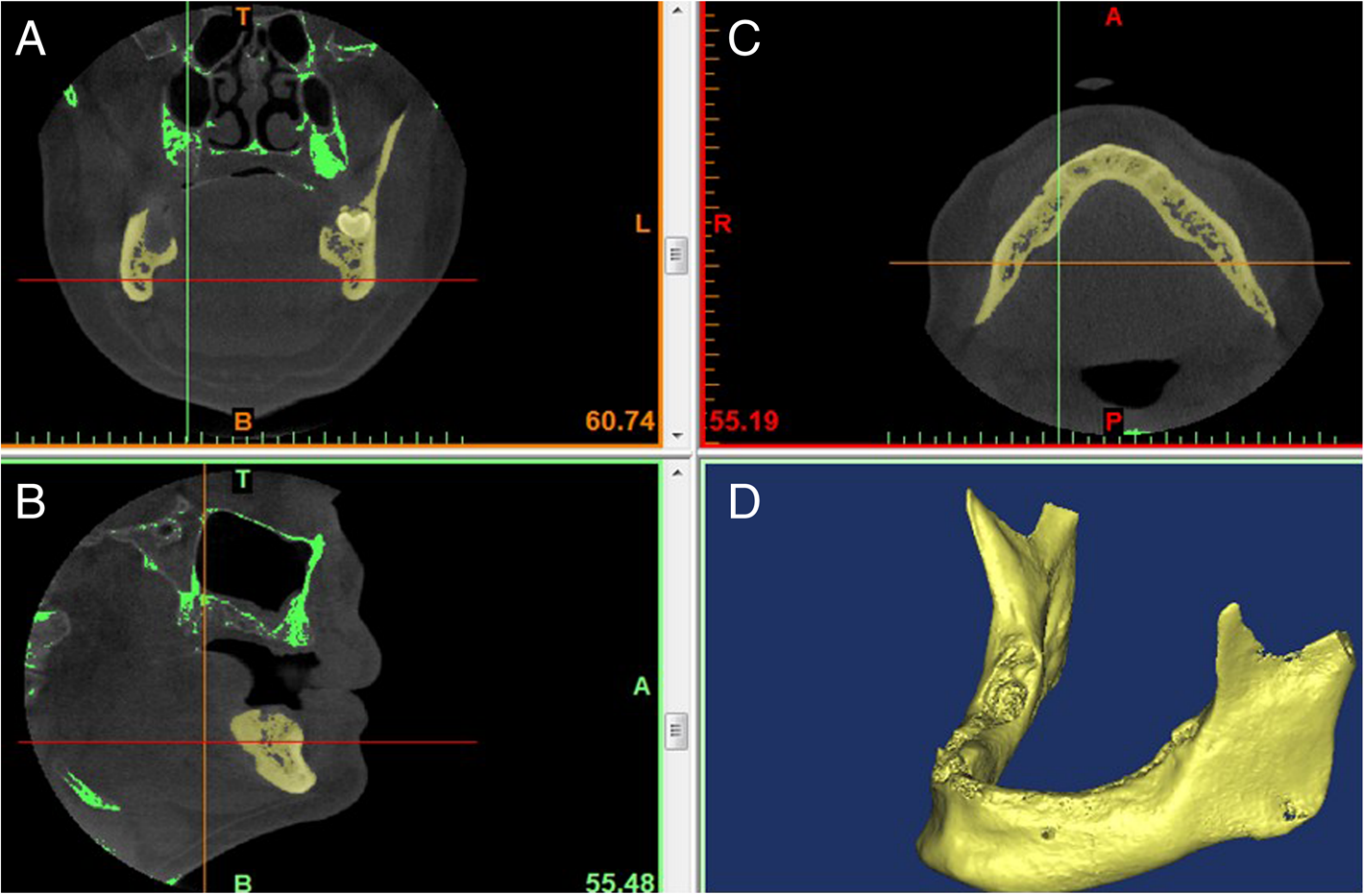
3D virtual model of the mandible reconstructed from CT images. (**a**) Coronal plane, (**b**) transverse plane, (**c**) sagittal plane, and (**d**) 3D view

A platform MIMICS (V16.0, Materialise, Belgium) was used to construct the 3D mandible based on the DICOM data files. The reconstruction procedure was as follows: first, a threshold for bony tissue segmentation was determined from the Hounsfield unit (HU) value and boundary. The mandible was separated as a sole mask through ROI (region of interest) extraction. Based on the mask, a 3D model represented as triangular mesh (also known as STL file) was created. Figure 2 shows the 3D model of the reconstructed mandible.

The triangular mesh is only a surface model. FEM calculation requires a volume mesh (tetrahedron) model. The MIMICS provides a mesh tool named 3-Matic (V8.0) for mesh generation and optimization. 3D virtual models can be smoothed and re-meshed to form volume meshes with high quality for numerical simulation. Geomagic (V12, 3D system, Rock Hill, SC, USA) software was used to complete the final editing of the volume mesh model. A portion of the reconstructed model was extracted for graft designs. The finalized volume mesh model in Geomagic program was directly imported to Abaqus (V6.13, Dassault System, Cedex, France) software for subsequent simulation and calculation.

### Material properties of mandible

Because of various degree of calcification of the mandible, different parts of the mandible have different elastic moduli and Poisson’s ratio. Therefore, a mandibular model should be treated as an inhomogeneous material. Calculations of elastic modulus of bone in relation to bone mass density and Hounsfield unit are expressed in the following equations [38]:

1 $$ \rho =114+0.756\times HU $$

2 $$ E=0.51\times {\rho}^{1.37} $$

where, *ρ* is the density, *HU* is the Hounsfield unit.; *E* is the modulus of elasticity.

### Mandibular graft design

**Fig. 3:**
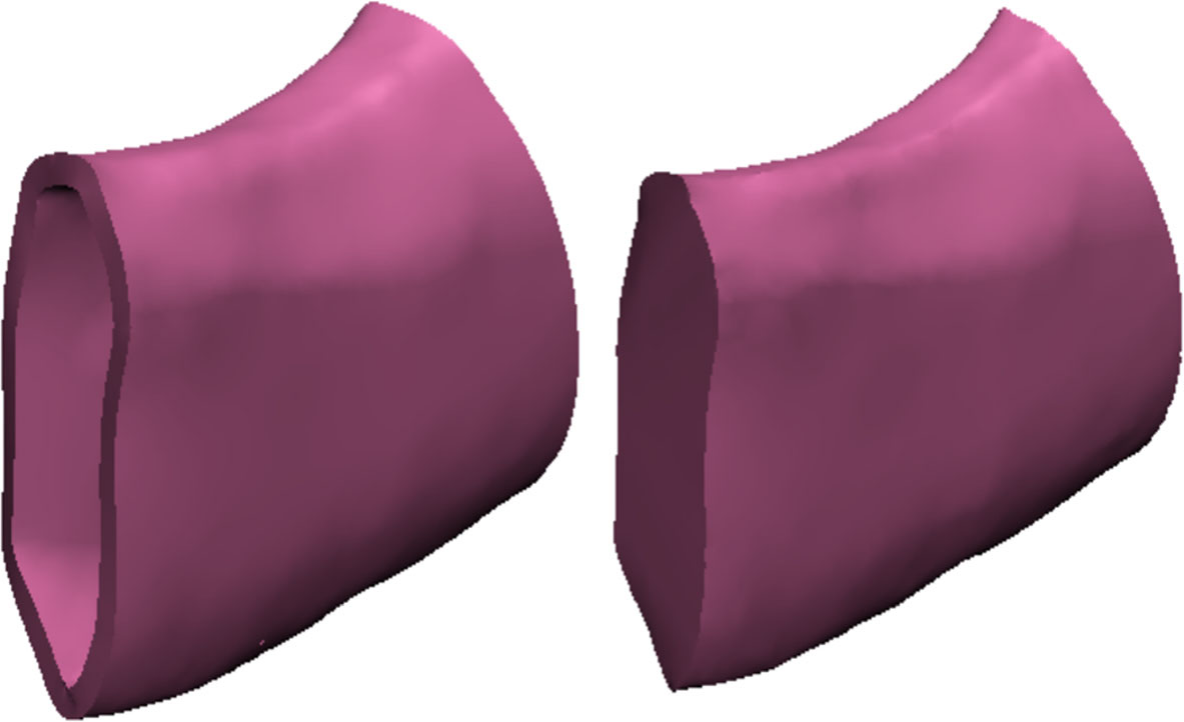
Reconstructed mandible shell (left) and core (right)

The models with high-quality volume meshes was exported as Abaqus file (.inp) for topological optimization. To compare the performance of the PLA printed grafts with original mandible, the bone material properties were also imported to Abaqus for FEA study. The reconstructed model was first split into two parts, shell and core, as shown in Fig. 3.

The shell was to maintain the original shape of the jawbone, and the thickness was set to 1.5 mm. The core was designed by topological optimization in Abaqus to form porous scaffold structure. Topology optimization is a mathematical method, which can optimize material layout and maximize system performance under given conditions. The mathematical model of the topological optimization can be expressed as:

Find:

3 $$ a={\left({a}_1,{a}_2,\cdots, {a}_n\right)}^T $$

Min:

4 $$ C(a)=\frac{1}{2}{F}^TM $$

5 $$ \mathrm{S}.\mathrm{t}.\left\{\begin{array}{c}\frac{V^{\ast }}{V}=p\\ {}F= NM\\ {}0<{a}_{\mathrm{min}}\le {a}_i\le 1\ \left(i=1,2,\cdots, n\right)\end{array}\right. $$

where, *a*_*i*_ is design variable whose value is continuous between (*a*_*min*_, 1) (*a*_*min*_ is a non-zero value to avoid singularity); *n* is the number of optimum design variable; *C(a)* is the global strain energy of the structure; *F* is the global vector of structural force; *M* is the global vector of structure displacement; *V* and V^*^ are the volume of structure before and after optimization; *p* is the prescribed volume fraction; *N* is the global stiffness matrix of the structure.

**Fig. 4:**
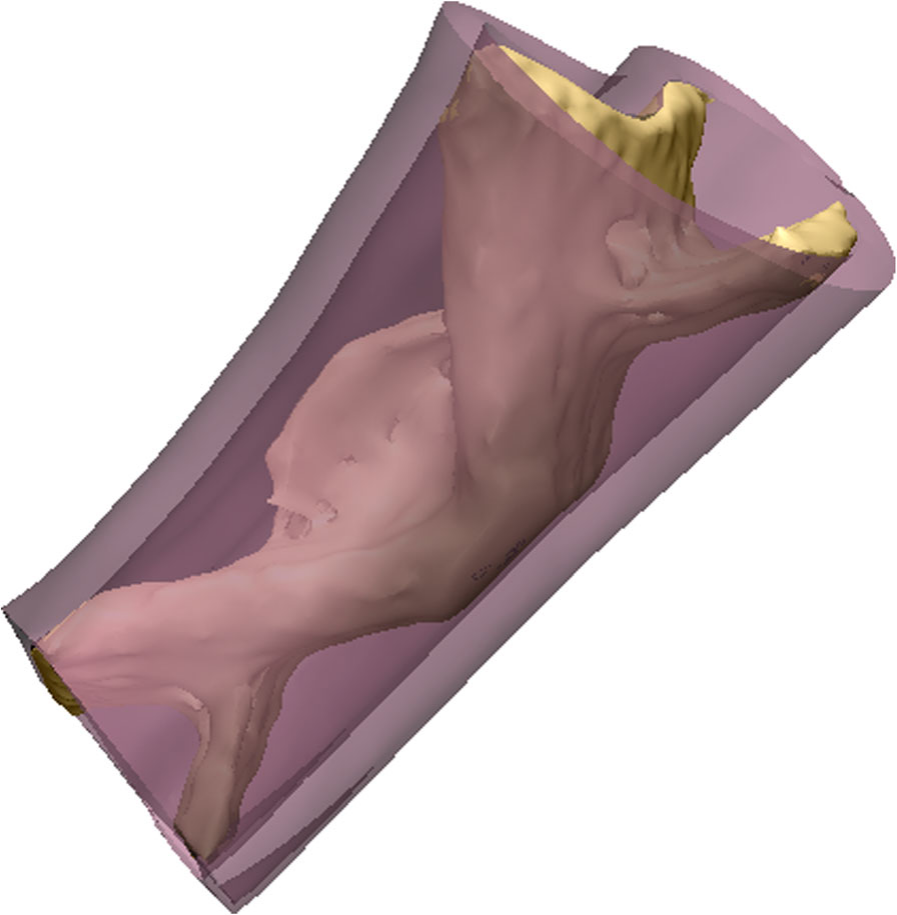
Graft with topological optimized-pore scaffold structure

The design of mandibular graft is shown in Fig. 4.

**Fig. 5:**
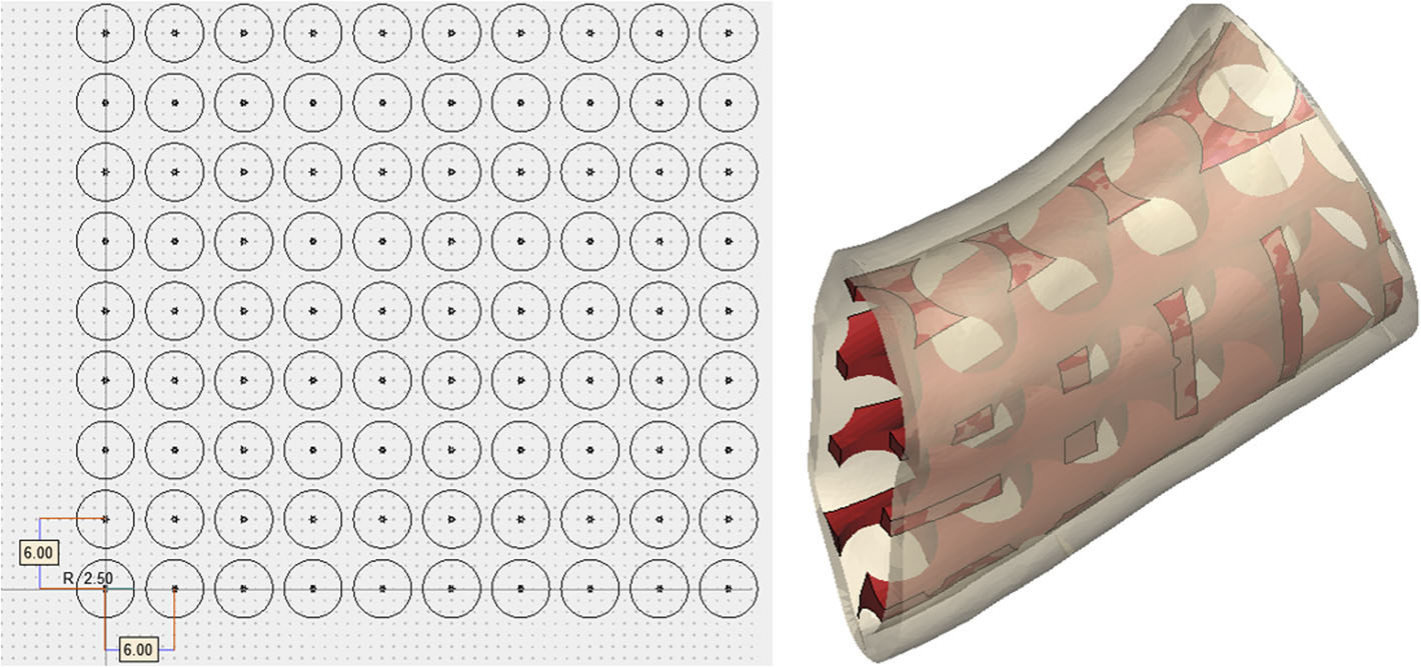
Graft with the round-pore scaffold structure

**Fig. 6:**
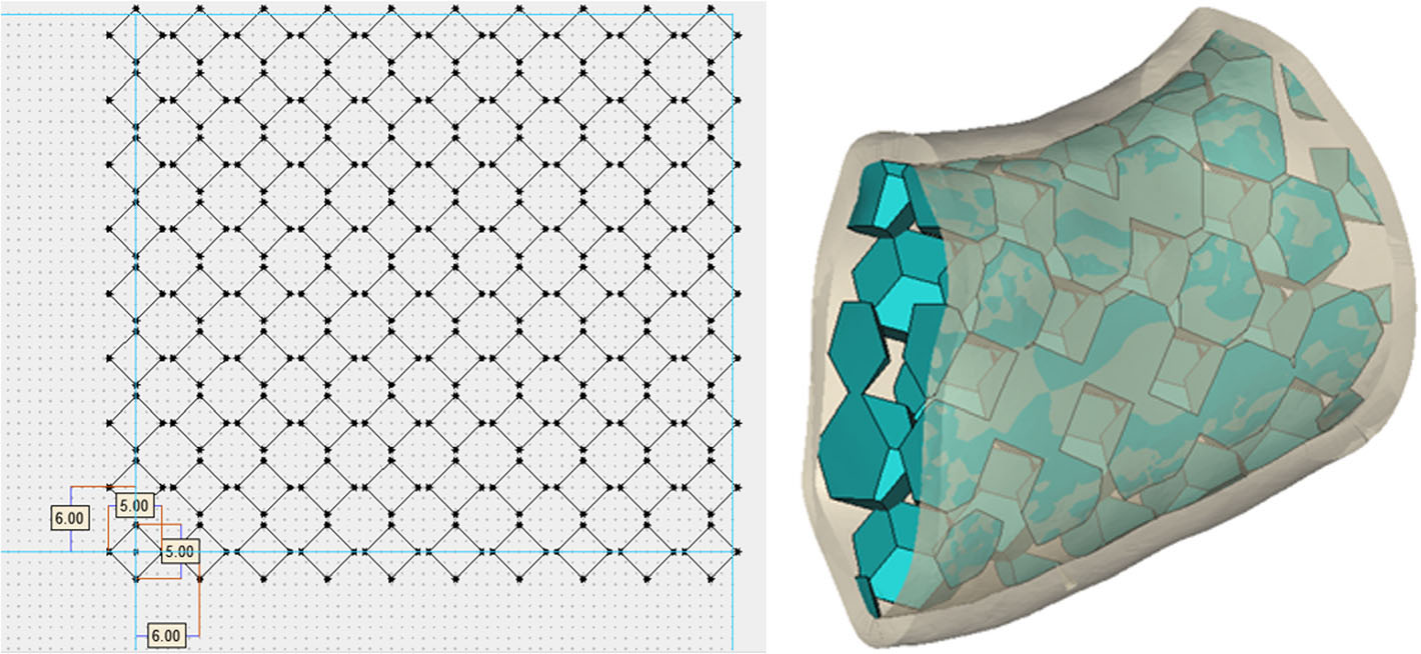
Graft with the square-pore scaffold structure

This study provided other two designed grafts with porous scaffold structure for comparison [39–42]. The porous scaffold structure contained distinct mesh configurations and a highly interconnected macroporous network. The macroporous structures were aligned with same intervals without going through the topology optimization process. Figure 5 shows the graft with round-pore struts. The radius of the circle was 2.5 mm, and the distance between adjacent circle centers was 6 mm. Figure 6 shows the graft with square-pore struts. The square diagonal was 5 mm, and the distance between adjacent square centers was 6 mm.

### 3D printing and anisotropic constitutive model

Grafts in this study were fabricated using an FDM 3D printer with a 210 × 150 × 100 mm build space (CEL Robox, UK). The grafts were fabricated layer-by-layer deposition of molten PLA material via FDM method. Each layer solidified at different intervals. The bonding between layers is relatively weaker than the material itself, causing the variation in the uniaxial tension strength with directions and anisotropic behavior. This study considered the effect of anisotropy of material on the mechanical behavior of graft, and proposed a constitutive model, which is expressed as follow:

6 $$ \left[\begin{array}{l}{\varepsilon}_{11}\\ {}{\varepsilon}_{22}\\ {}{\varepsilon}_{33}\\ {}{\gamma}_{12}\\ {}{\gamma}_{13}\\ {}{\gamma}_{23}\end{array}\right]=\left[\begin{array}{cccccc}\frac{1}{E_1}& -\frac{v_{21}}{E_1}& -\frac{v_{31}}{E_3}& 0& 0& 0\\ {}-\frac{v_{12}}{E_1}& \frac{1}{E_1}& -\frac{v_{32}}{E_3}& 0& 0& 0\\ {}-\frac{v_{13}}{E_1}& -\frac{v_{23}}{E_1}& \frac{1}{E_3}& 0& 0& 0\\ {}0& 0& 0& \frac{1}{G_{12}}& 0& 0\\ {}0& 0& 0& 0& \frac{1}{G_{13}}& 0\\ {}0& 0& 0& 0& 0& \frac{1}{G_{23}}\end{array}\right]\left[\begin{array}{l}{\sigma}_{11}\\ {}{\sigma}_{22}\\ {}{\sigma}_{33}\\ {}{\tau}_{12}\\ {}{\tau}_{13}\\ {}{\tau}_{23}\end{array}\right] $$

where,1, 2 and 3 are the principal axes directions; ɛ is the normal strain; γ is the shear strain; σ is the normal stress; τ is the shear stress; *E* is the Young’s modulus; *v* is the Poisson’s ratio; *G* is the shear modulus of the material.

The Young’s and shear moduli of the two perpendicular directions, and the corresponding Poisson’s ratios are the same. Thus, the material comes close to being a transversely isotropic material. Transverse isotropy is a special subclass of orthotropic and characterized by a plane of isotropy at every point in the material. Assuming 1–2 plane is the plane of isotropy at every point, transverse isotropy requires *E*_1_ = *E*_2_ = *E*_*p*_, *v*_31_ = *v*_32_ = *v*_*tp*_, *v*_13_ = *v*_23_ = *v*_*pt*_, *G*_13_ = *G*_23_ = *G*_*t*_*,* where *p* and *t* stand for “in-plane” and “transverse,” respectively. Thus, *v*_*tp*_ has the physical interpretation of the Poisson’s ratio that characterizes the strain in the plane of isotropy resulting from stress normal to it. *v*_*pt*_ characterizes the transverse strain in the direction normal to the plane of isotropy resulting from stress in the plane of isotropy. The stress-strain laws reduce to

7 $$ \left[\begin{array}{l}{\varepsilon}_{11}\\ {}{\varepsilon}_{22}\\ {}{\varepsilon}_{33}\\ {}{\gamma}_{12}\\ {}{\gamma}_{13}\\ {}{\gamma}_{23}\end{array}\right]=\left[\begin{array}{cccccc}\frac{1}{E_p}& -\frac{v_p}{E_p}& -\frac{v_{tp}}{E_t}& 0& 0& 0\\ {}-\frac{v_p}{E_p}& \frac{1}{E_p}& -\frac{v_{tp}}{E_t}& 0& 0& 0\\ {}-\frac{v_{pt}}{E_p}& -\frac{v_{pt}}{E_t}& \frac{1}{E_3}& 0& 0& 0\\ {}0& 0& 0& \frac{1}{G_p}& 0& 0\\ {}0& 0& 0& 0& \frac{1}{G_t}& 0\\ {}0& 0& 0& 0& 0& \frac{1}{G_t}\end{array}\right]\left[\begin{array}{l}{\sigma}_{11}\\ {}{\sigma}_{22}\\ {}{\sigma}_{33}\\ {}{\tau}_{12}\\ {}{\tau}_{13}\\ {}{\tau}_{23}\end{array}\right] $$

where

$$ {G}_p={E}_p/2\left(1+{v}_p\right). $$

**Fig. 7:**
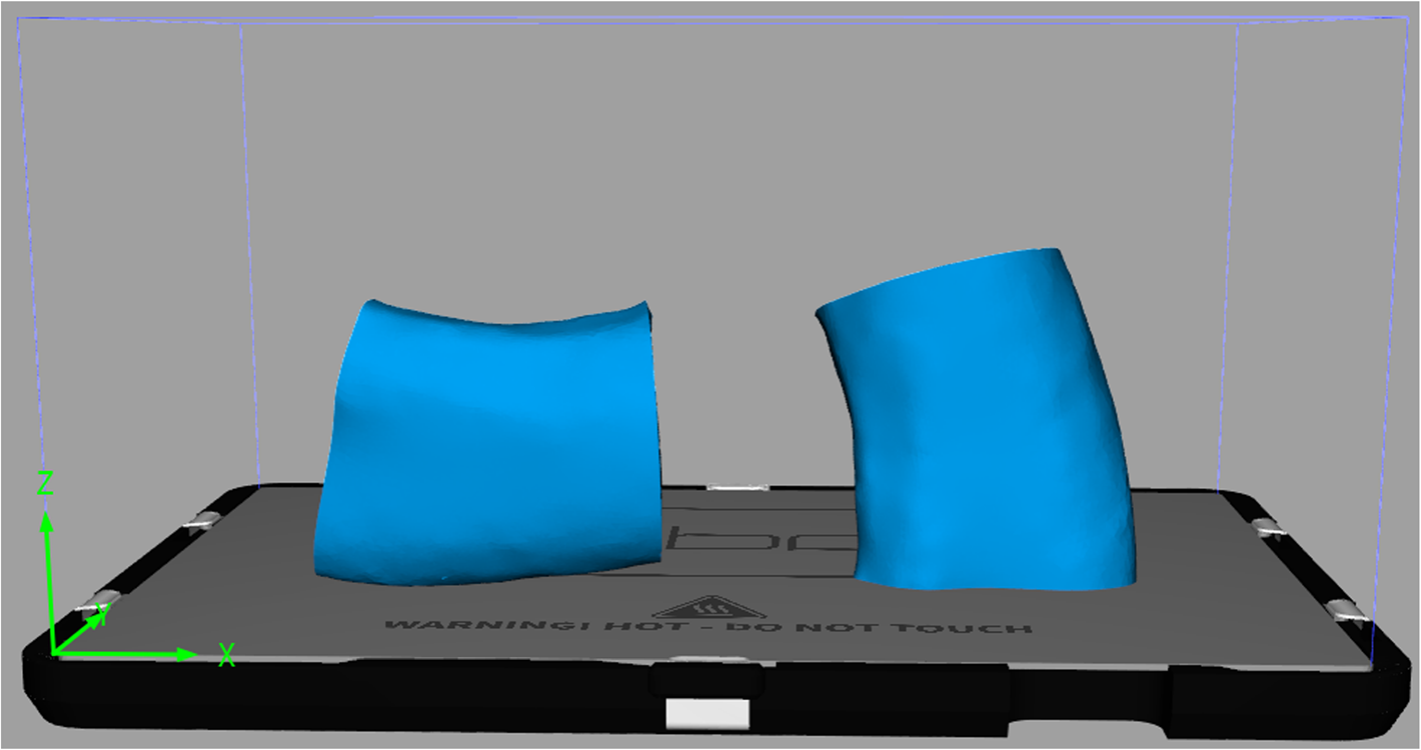
Schematic of mandibular grafts with printing angle 0° (left) and 90° (right)

The total number of independent parameters was only five. These parameters were obtained by designing a three-point bending test of 3D printed beam specimens. To evaluate the effect of printing direction on mechanical behavior, all three designed graft models were imported into the 3D printer by STL file format and printed with 0, 45 and 90 degrees orientations with PLA material. Figure 7 shows the graft models with different printing angle. The nozzle diameter was set as 0.4 mm while the layer thickness was set as 0.1 mm. According to the printed specimens, the resolution of the printer was sufficient to print the designed microstructure of the graft. Some post-processing such as polish, cutting and support removing were also conducted.

### Three-point-bending test

**Fig. 8:**
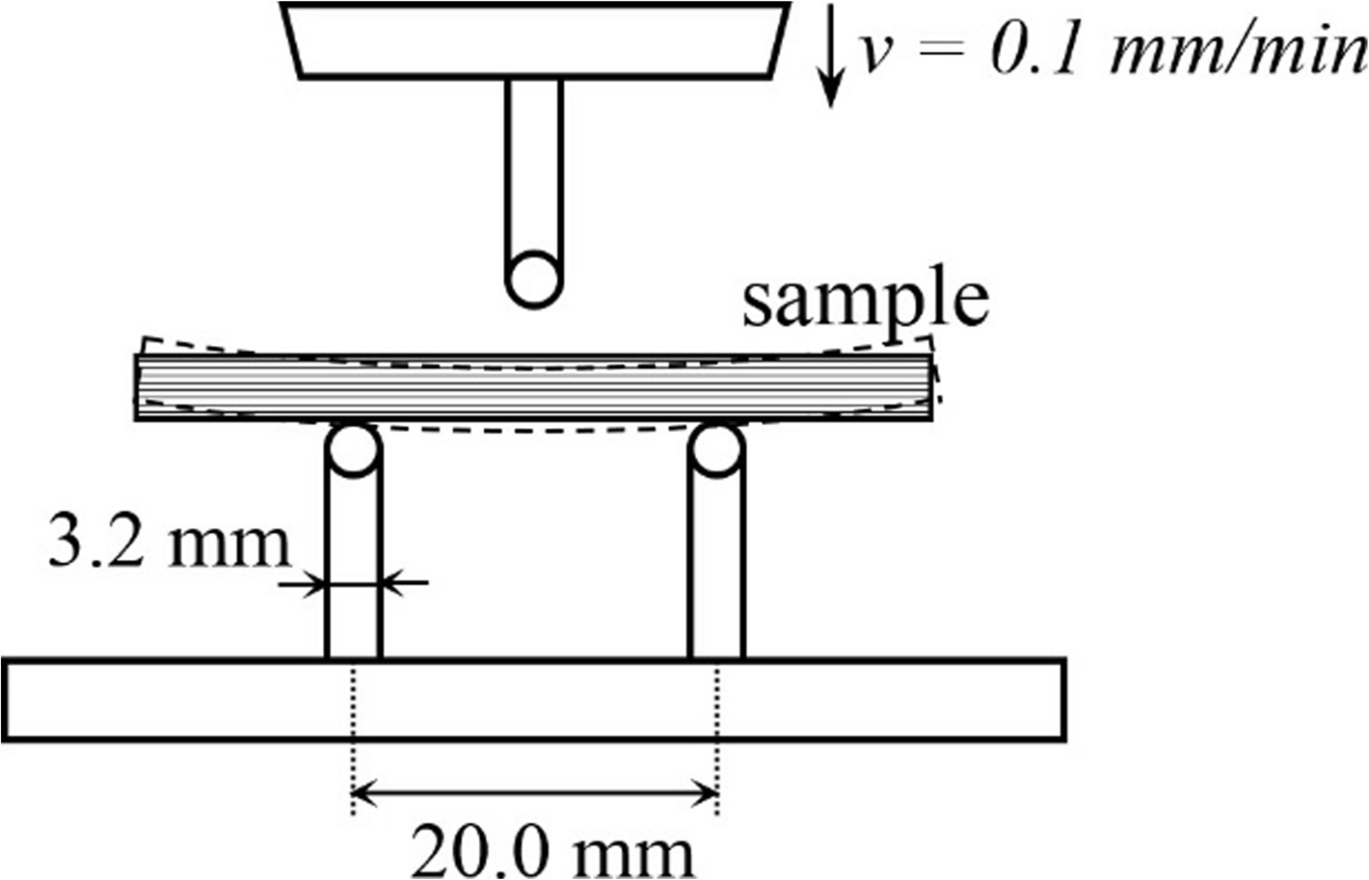
Setup of three-point bending test

A three-point-bending jig was used to evaluate mechanical behaviors of the printed beams. The setup of the three-point bending test is shown in Fig. 8. The lower support spans were 20 mm apart and the upper loading nose was set an equal distance from each span. Two supporting rods and the loading nose had the same radius of 3.2 mm. 0.1 mm/min was set for the loading velocity.

The beam specimens with different printing angle 0°, 45° and 90° were tested. The 0-degree specimens had printing angle perpendicular to the loading cross-section; the 45-degree specimens had a 45° angle between loading cross-section and printing direction, and the 90-degree specimens had a printing angle parallel to the loading section. Three duplicate specimens were prepared for each printing angle with the same dimension. The dimensions were 30 mm × 10 mm × 5 mm. The mid-span flexural stress and strain were calculated according to formulas provided by ASTM standard [43].

8 $$ {\sigma}_f=\frac{3 PL}{2{bd}^2} $$

9 $$ {\varepsilon}_f=\frac{6 Dd}{L^2} $$

where, *σ*_*f*_ is the flexural stress; *P* is the load at the center of the beam; *L* is the support span; *b* is the width of beam tested; *d* is the depth of beam tested; *ɛ*_*f*_ is sample strain; *D* is the deflection at the center of the beam.

In addition to the beam samples, the effect of printing angle (0^0^, 45^0^ and 90^0^) of grafts on mechanical strength was also evaluated by a three-point bending test. Four grafts of each printing type were prepared and tested. Maximum load, field load, failure deflection and yield deflection were measured and the biomechanical behavior of each group was compared.

### Finite element analysis

**Fig. 9:**
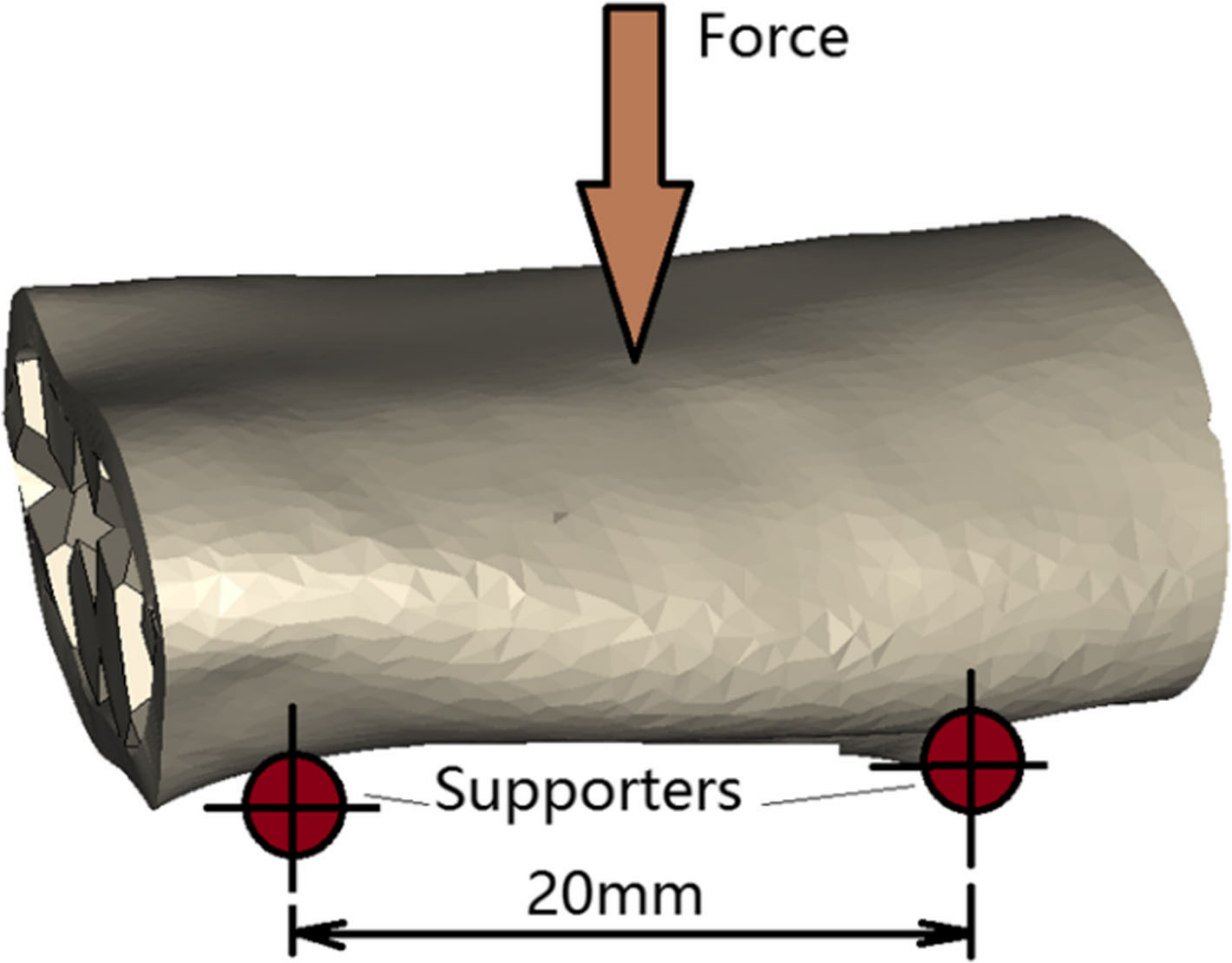
Setup of grafts subjected to three-point bending load

FEA calculations are based on finite element models that allow the validation of the experimental observations and calibrate the constitutive relationship between virtual and physical models [44]. The FE models were built with the material parameters obtained from the three-point bending test described in 2.5 using Abaqus. The transverse anisotropic material models were used with the principal axis set based on the printing directions. The digital grafts with different printing directions were subjected to loads corresponding to the three-point bending test. The initialization and propagation of cracks at the bottom were modeled with the extended finite element method (XFEM). The schematic setup of grafts subjected to loading is shown in Fig. 9. The mechanical responses were obtained from the computational simulations.

## Results

### Three-point-bending test of beam specimens

**Fig. 10:**
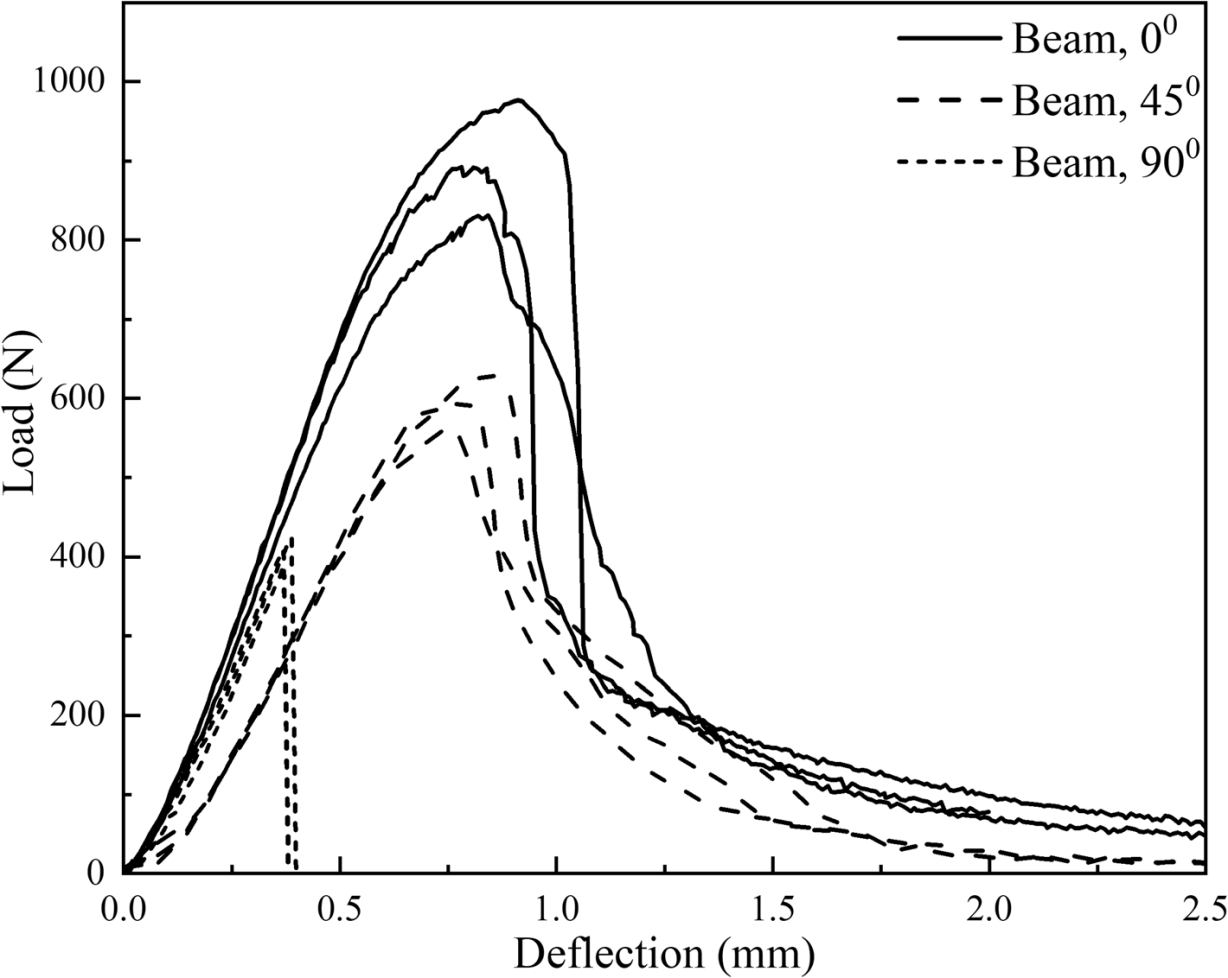
Load-deflection curves of three-point bending test on beam specimens

**Fig. 11:**
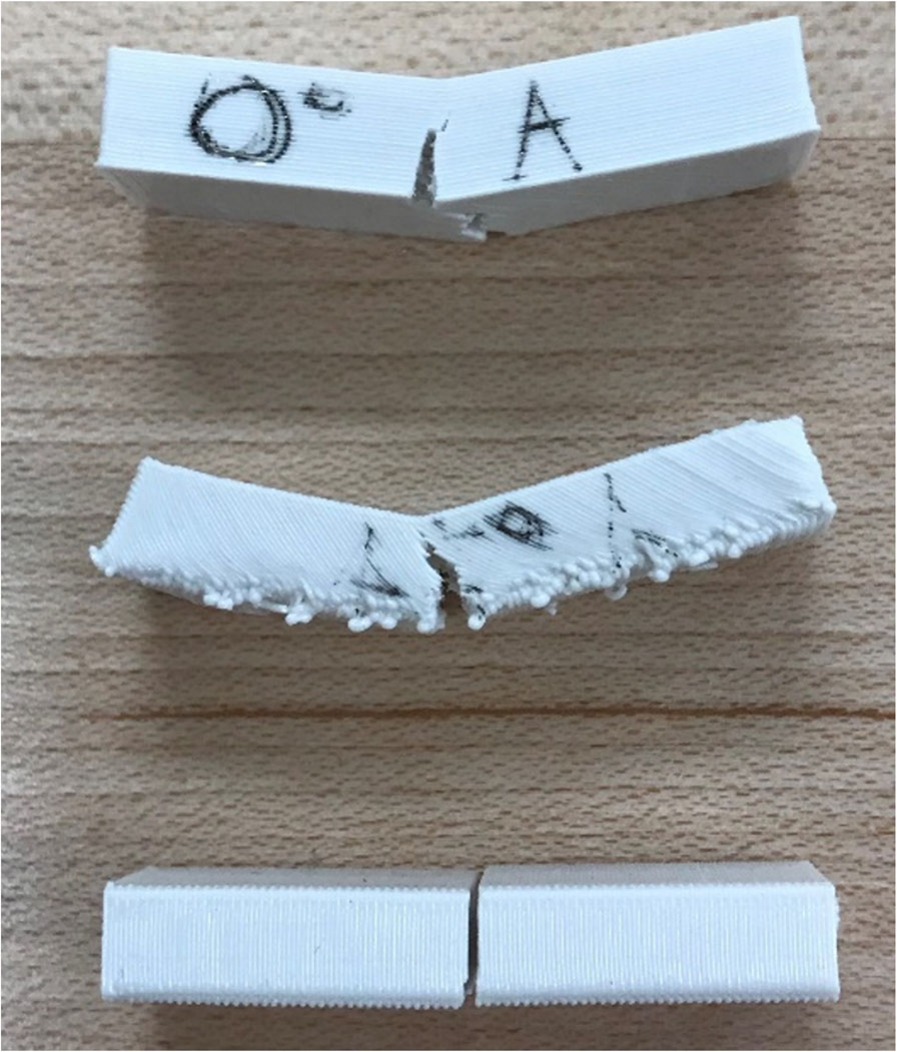
Failure mode of three beam specimens with printing angle 0^0^, 45^0^, 90^0^ (from top to bottom)

Experimental data of the three-point bending test on beams were summarized in Fig. 10. The results show good repeatability of samples in the same group. The yield stress, failure stress, failure strain, ultimate strength and flexural modulus were calculated for each sample to better understand the mechanical performance of the printed material. The yield stress was calculated using the method of 0.2% offset; failure stress corresponds to the stress at failure strain, ultimate strength obtained from the maximum stress value. Flexural modulus was along the linear portion of the stress-strain curve. All these five properties were evaluated for each printed graft. The average value and standard deviation of each group are summarized in Table 1. Figure 11 shows a typical failure mode and crack distribution of beam specimens from each group.

**Table 1:** Flexural properties of PLA beam specimens with three printing conditions

Printing Angle (degree)	Ultimate Strength (MPa)	Yield Strength (MPa)	Failure Strength (MPa)	Failure Strain (%)	Flexural Modulus (GPa)
0	107.98 (7.13)^a^	94.68 (7.90)	96.60 (10.00)	7.36 (0.34)	2.06 (0.08)
45	71.53 (3.45)	69.70 (3.73)	68.26 (2.80)	5.77 (0.47)	1.21 (0.03)
90	49.20 (1.71)	49.20 (1.71)	49.20 (1.71)	2.82 (0.08)	1.59 (0.07)

^a^Values in parentheses are the standard deviations of each group

Based on the experimental results and computational model calibration, the following values of mechanical parameters were obtained for the anisotropic constitutive model (in the printing plane: *E*_1_ = *E*_2_ = 1590 *MPa*, *G*_12_ = 722 *MPa*, perpendicular to the printing plane: *E*_3_ = 2050 *MPa*, *G*_23_ = *G*_13_ = 560 *MPa*, *v*_12_ = *v*_13_ = *v*_23_ = 0.42.).

### The three-point bending test of grafts

**Fig. 12:**
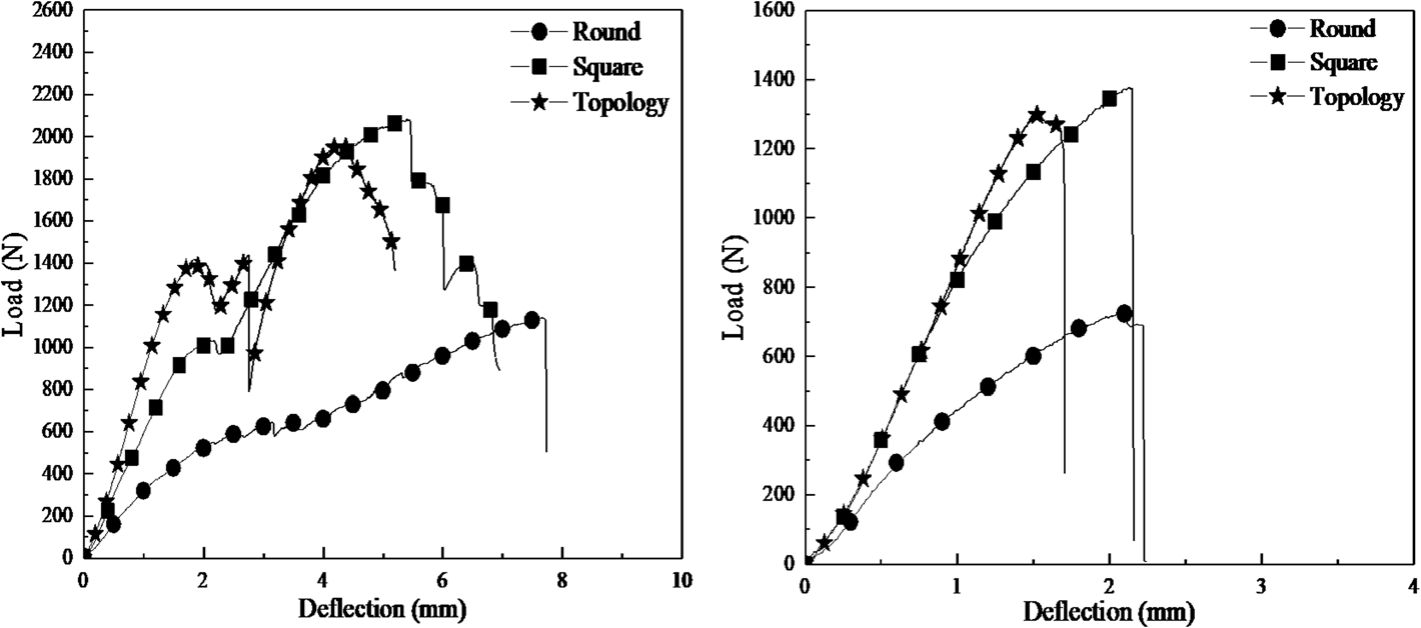
Load-deflection curves of three-point bending test on grafts printed at 0-degree (left) and 90-degree (right)

**Fig. 13:**
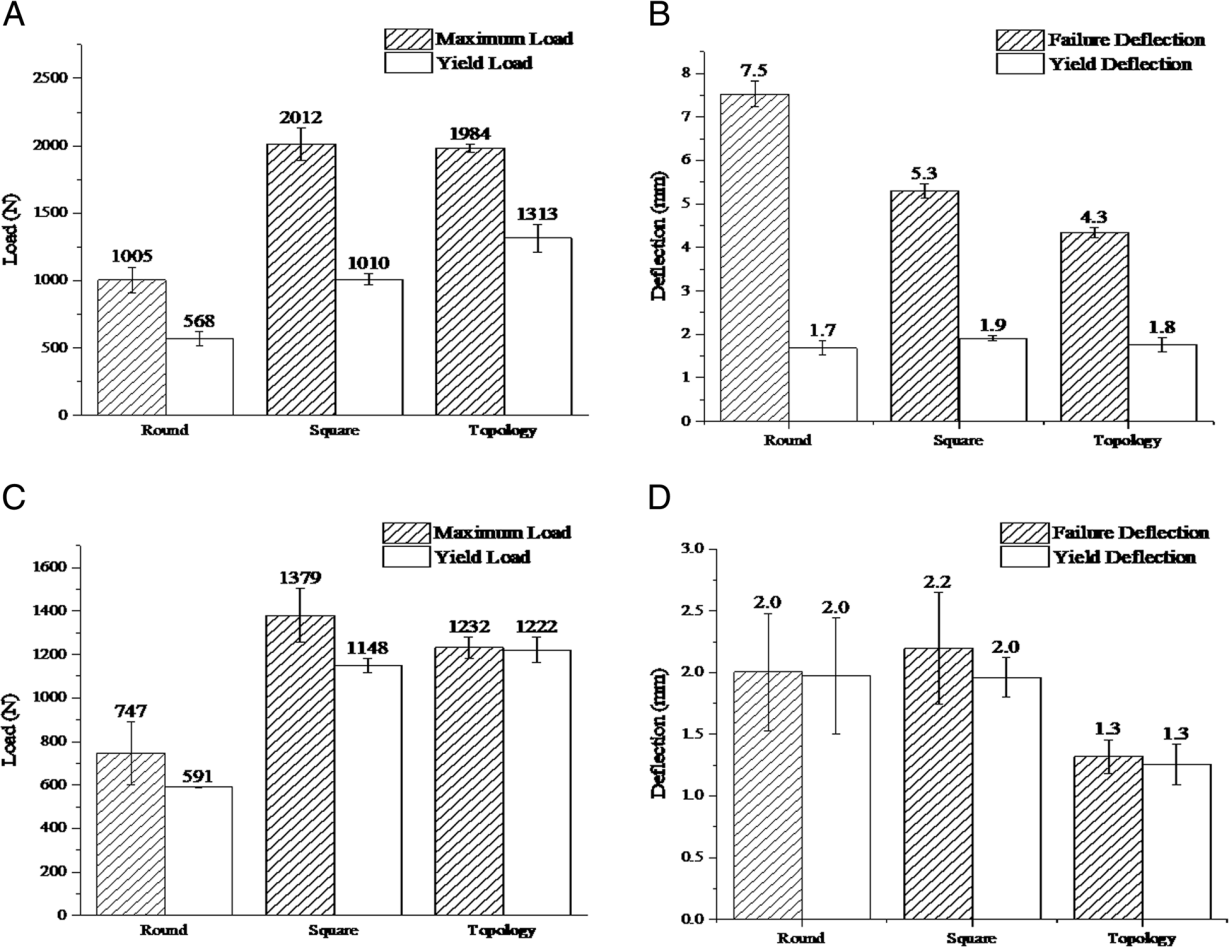
Histograms of flexural properties for PLA grafts. **a**. Load of specimens printed at 0-degree. **b**. Deflection of specimens printed at 0-degree. **c**. Load of specimens printed at 90-degree. **d**. Deflection of specimens printed at 90-degree

**Fig. 14:**
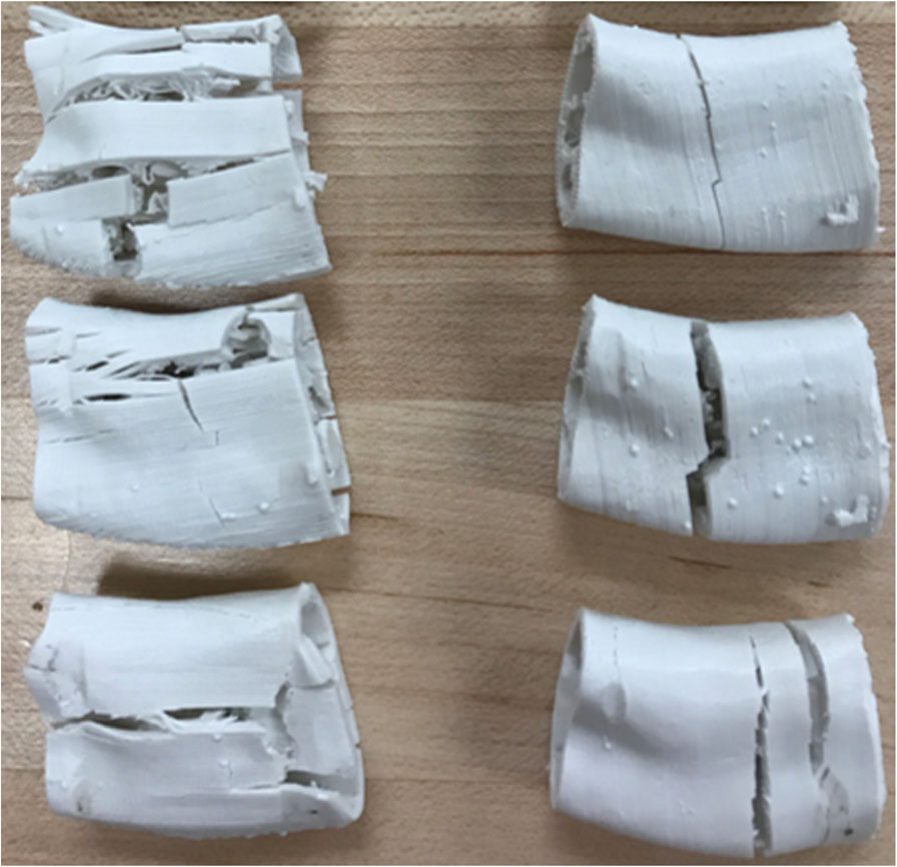
Comparison of failed grafts with round-pore, square-pore and topological optimized-pore scaffold structures, from top to bottom printed at 0-degree (left) and 90-degree (right)

The results of three-point bending test from different types of printed grafts are summarized in Fig. 12. The “Round”, “Square” and “Topology” indicate the grafts with round-pore internal structure, square-pore structure, and topological optimized-pore internal structure, respectively. The effect of printing angle on mechanical behavior of maximum load, yield load, failure deflection and yield deflection were calculated and compared for each group, as shown in Fig. 13. Figure 14 shows the failure mode and crack propagation of different grafts printed with 0 and 90 degree angles. Three grafts on the left in Fig. 14 are the 0-degree printed samples while the three right samples were printed at 90-degree.

### The finite element analysis of grafts

**Fig. 15:**
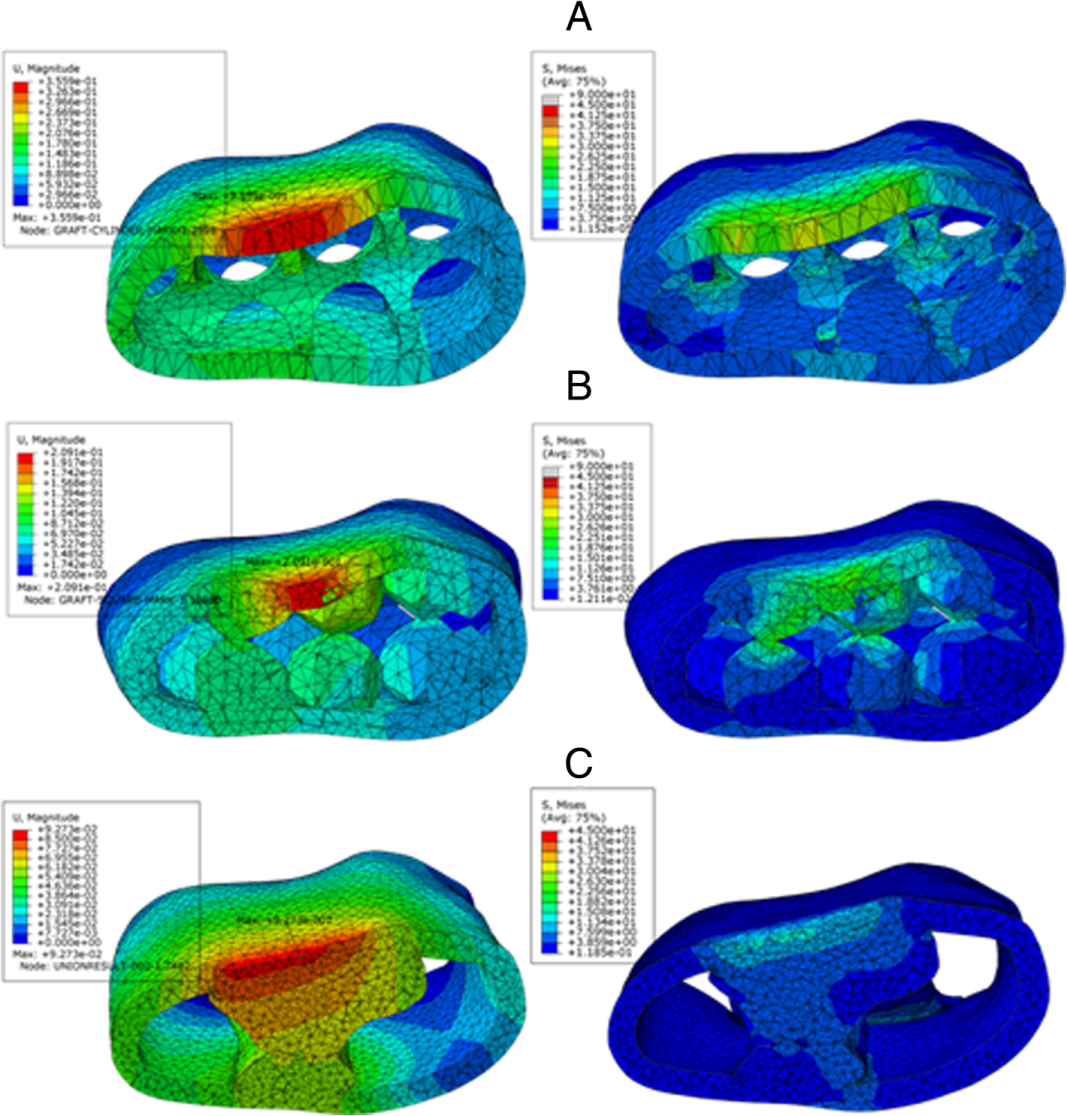
FEA results of grafts. **a**. Round-pore. **b**. Square-pore. **c**. Topological optimized-pore internal scaffold

Figure 15 is the cross-sectional FEA results, which provide detailed information on maximum displacement, and Von Mises stress distribution of the three porous structures of grafts. For each graft, the left side displays the maximum displacement near the loading nose location and the global maximum displacement spot. The right side graph displays the Von Mises stress distribution. Since the anisotropic constitutive model proposed in this study is based on the linear-perfect plastic model which is insufficient to capture the plastic stage and large deformation, all computational models were subjected to a 500 N three-point bending load that represents maximum chewing force from patients.

Table 2 lists the maximum principal strain, maximum Von Mises stress, maximum displacement and porosity of each design of graft. Porosity is a measure of the void spaces in a graft and is a fraction of the volume of voids over the total volume based on the reconstructed mandibular core scaffold. Porosity can be theoretically calculated from the 3D computational model and real porosity can be calculated from printed graft using Archimedes principle that the volume of displaced water is equal to the volume of the submerged object. When printing some models with spatial structure, like graft in this case, the printer will generate some supports to avoid structural collapse, and some supports are difficult to remove after printing. Therefore, the printed porosity is often slight smaller than designed porosity. However, the error in this study is below 5%, which is acceptable.

**Table 2:** Flexural properties for the PLA grafts

Porous Structure	Max Principal Strain (%)	Max Von Mises Stress (MPa)	Max Displacement (mm)	Theoretical Porosity (%)	Real Porosity (%)
Round	1.972	49.386	0.356	77	75 (3)
Square	1.077	25.045	0.208	58	55 (2)
Topology	0.533	17.331	0.093	67	65 (2)

^a^Values in parentheses are the standard deviations of each group

## Discussion

PLA has not been currently used as bone analogs for mandibular reconstruction surgery in load-bearing areas. Its excellent biocompatibility and biodegradation properties are important reason for extensively studied in the literature as a scaffolding material for tissue engineering in craniofacial areas. Further strategies to improve its mechanical properties based on PLA modifications and PLA nanocomposite designs may be the key to improve its mechanical properties for mandibular reconstruction. This project is to provide valid information for readers to understand mechanical issues of 3D printed biomaterials.

### Comparison of 3D printed beams with different printing angles

The load-deflection curves of Fig. 10 from three-point bending test validate that the direction of printing angle has a substantial effect on the mechanical properties of 3D printed parts. From the load-deflection curves of beam printed at 0-degree angle, the beam showed its elastic property in the initial loading stage. With the load increased, the beam entered the plastic stage, and plastic strain developed and cracked until global damage. The similar trend was observed from the load-deflection curves of beam printed at 45-degree angle. However, the load-deflection curve for beam specimen printed at 90-degree angle doesn’t show apparent yield or horizontal segment. After reaching the peak point, the crack propagates suddenly penetrated through the whole cross-section and the beam fractured into two parts. This is different from the flexural curves for the specimens printed at 0-degree or 45-degree angles, which show slight plastic failure before their rupture occurred.

Figure 11 shows the beam specimens with 0-degree printing angle presents flexural failure and the main cracks appeared in the mid-span and prorogated along the middle axis. The same failure mode can be seen in the beam specimens printed at 45-degree angle with cracks grew along 45-degree printing direction. For the beam specimens printed at 90-degree angle, it presents shear failure with the shear force exceeded the bond strength between the layers. The local failure and global failure occurred at a similar load. The beam was sheared off in the middle of the beam where the highest stress was concentrated.

The data from the three-point bending test of PLA (Table 1) also illustrate that all of the properties exhibit anisotropic behavior with property differences up to 60%. The average ultimate strength of a specimen printed at 0-degree was 54% higher than that of a specimen printed at 90-degree and 34% higher than that of those specimens printed at 45-degree. Similar patterns occurred on the failure stress and yield strength among those three groups. Furthermore, the average strain at failure was almost 2.5 times for the specimen printed at 0-degree than that printed at 90-degree and was also 14% higher than that printed at 45-degree. There was a great effect of printing direction on flexural modulus shown in Table 1. Flexural modulus values of specimens printed at 0-degree were 23% higher than that printed at 90-degree and 41% higher than specimens printed at 45-degree. Overall, beam specimens printed at 0-degree clearly performed the best among the three groups.

### Comparison of 3D printed grafts with different printing angles

The load-deflection curves from three-point bending test (Fig. 12) illustrate anisotropic behavior of printed grafts. Similar mechanical behavior trends are observed, i.e., elastic and plastic deformation, of grafts based on printing directions. The data of flexural properties for grafts (Fig. 13) show that the maximum load of each graft printed at 0-degree is much higher than that of 90-degree printed with differences up to 72%. The average failure deflection of grafts printed at 0-degree was much higher than that of 90-degree printed with differences up to 73%. The yield load and deflection are not significantly affected by printing angle. By combining the experimental data and failure mode of grafts, it is found that for grafts printed at 0-degree, local failure started from upper filament layers, the propagation of cracks was perpendicular to the direction of loading. As the deflection increased, the tensile force between the bottom filament layers exceeded the bond strength; the crack opening began to grow along the printing direction until the global failure occurred. On the contrast, for grafts printed at 90-degree, local failure began from the upper filament and then propagated along the loading direction, the global failure occurred at almost the same time since the bonding between the adjacent filaments was weak, and the cracks could easily penetrate through the whole filament layers. It explains why the direction of main crack propagation in Fig. 14 are different from grafts printed with 0^0^ and 90^0^. Also, it matches with the obtained results that the maximum load and deflection for grafts printed with 0^0^ are much higher than that of grafts printed with 90^0^.

### Comparison of the topological optimized graft with other designed grafts

The results of the finite element analysis of mechanical behaviors of the printed grafts are presented in Table 2. The maximum principal strain (%), Von Mises stress (MPa), and displacement (mm) of the topological optimized grafts decreases 73%, 65%, and 74% respectively when compared to those with round-pore structure design. Comparing to the square-pore structure, the optimized structure has a decrease of 51% in maximum principal strain, 31% in maximum stress, and 55% in displacement. The maximum Von Mises stress distribution of grafts (Fig. 15) indicates that the stresses are always concentrated at the top aspect of the graft. The same pattern is observed with maximum displacement. The topological optimized graft has significantly better results as most of the meshes keep very small stress and the maximum stress is well below the allowable stress (σ = 90 MPa). In order to compare the mechanical behavior of designed grafts with human natural mandible, the model of original structure of the mandible was built with the material parameters obtained from the CBCT files described in 2.2 under using Abaqus. The maximum principal strain, maximum Von Mises stress, and maximum displacement of original mandible model are 0.232%, 15.427 MPa, and 0.090 mm respectively. The topological optimized graft has the mechanical properties close to the real bone in terms of the maximum Von Mises stress and maximum displacement. The maximum principal strain of the optimized graft is slightly higher than that of the human mandible, which may be led by the difference of Young’s modulus and density between the bone and PLA material. The average Young’s modulus and density of bone in this patient are 10GPa and 1500 kg/m^3^, which is much higher than that of the PLA materials with 2GPa and 1200 kg/m^3^. However, the mechanical properties of the other two designed grafts are relatively poor, which cannot provide a good environment for defect repair in clinic.

The real porosity (%) of the topological optimized graft is 65, which increases 15% when compared to the grafts with square-pore structure. Although the porosity of topological optimized graft is lower than graft with round-pore scaffold structure, the strength and stiffness are significantly improved. The results verify that the topological optimization method is capable of optimizing the grafts by assigning more strut elements to the stress concentration area and removing strut elements from the low-stress area. In this way, graft can have higher space utilization, higher strength and stability, and more uniform stress distribution. However, these two conventional grafts use uniform configuration of strut elements, which can cause stress concentration and material waste. Overall, the printed graft designed by topological optimization method provides high strength, stiffness, and porosity that can provide a conducive environment from mechanical and biological perspectives.

## Conclusions

A new method of restoring segmental bony defects of the mandible to its exact original shape, size and form is proposed by using 3D printing technology and topological optimization method. 3D printing technology can easily fabricate complex shapes to match patients’ unique defects. The 3D printed graft samples had anisotropic properties. Printing direction and internal design of the grafts significantly affected their mechanical properties. The grafts printed at 0° with topology optimization had the best results. Although the results of this study are based on PLA material, the proposed methodologies are also applicable to other promising 3D printing materials such as Polyetheretherketone (PEEK). 3D printing technology and topological optimization are useful tools in fabrication and designing bone analogs for mandibular reconstruction.

## Abbreviations

ABS: Acrylonitrile butadiene styrene
CBCT: Cone beam computed tomography
FDM: Fused deposition modeling
FEM: Finite element modeling
PLA: Polylactic Acid
